# Impact on early outcomes and immune reconstitution of high-dose post-transplant cyclophosphamide vs anti-thymocyte globulin after reduced intensity conditioning peripheral blood stem cell allogeneic transplantation

**DOI:** 10.18632/oncotarget.24328

**Published:** 2018-01-27

**Authors:** Christelle Retière, Catherine Willem, Thierry Guillaume, Henri Vié, Laetitia Gautreau-Rolland, Emmanuel Scotet, Xavier Saulquin, Katia Gagne, Marie C. Béné, Berthe-Marie Imbert, Beatrice Clemenceau, Pierre Peterlin, Alice Garnier, Patrice Chevallier

**Affiliations:** ^1^ Etablissement Français du Sang, Nantes, France; ^2^ CRCINA, INSERM, CNRS, Université d’Angers, Université de Nantes, Nantes, France; ^3^ Hematology Department, CHU, Nantes, France; ^4^ LabEx Transplantex, Université de Strasbourg, France; ^5^ Hematology/Biology Department, CHU, Nantes, France; ^6^ INSERM, Centre de Recherche en Transplantation et Immunologie, UMR1064, Université de Nantes, Nantes, France; ^7^ Service de Virologie, CHU Nantes, Nantes, France; ^8^ LabEx IGO “Immunotherapy, Graft, Oncology”, Nantes, F-44000, France

**Keywords:** allogeneic bone marrow transplantation, post-transplant cyclophosphamide, immune reconstitution, immunology

## Abstract

We have compared prospectively the outcome and immune reconstitution of patients receiving either post-transplant cyclophosphamide (PTCY) (*n* = 30) or anti-thymocyte globulin ATG (*n* = 15) as Graft-versus-host disease (GVHD) prophylaxis after reduced-intensity conditioning (RIC) allogeneic peripheral blood stem cell (PBSC) transplantation (allo-SCT). The outcome and immune reconstitution of patients receiving either of these two regimens were compared prospectively. This study allowed also to investigate the impact of PTCY between haplo-identical vs matched donors and of clofarabine as part of the RIC regimen. The γ/δ T-cells, α/β T-cells (CD8^+^ and CD4^+^), NK T-cells, NK cells, B-cells, Tregs and monocytes were analyzed by flow cytometry from a total of 583 samples. In the PTCY group significant delayed platelets recovery, higher CD3+ donor chimerism, higher HHV-6 and lower EBV reactivations were observed. Early survival advantage for CD4+ T-cells, Tregs and α/β T-cells was documented in the PTCY group while it was the case for α/β T-cells, NK cells and monocytes in the ATG group. Higher counts of NK and monocytes were observed at days +30 and/or day+60 in the ATG group. Both results were retained even in the case of mismatched donors. However, higher percentages of CD4+ T-cells, α/β T-cells and Tregs were observed with haplo-identical donors in the PTCY group. Finally, clofarabine was responsible for early survival advantage of NK T-cells in the PTCY group while it abrogated the early survival advantage of γ/δ T-cells in the ATG group. In conclusion, there are marked differences in the immunological effects of ATG vs PTCY as GVHD prophylaxis for RIC PBSC allo-SCT.

## INTRODUCTION

After the revolution that has constituted almost twenty years ago the introduction of reduced intensity conditioning (RIC) regimens, allowing to perform allogeneic stem cell transplantation (allo-SCT) in older patients or patients with co-morbidities, [1–3] a new step has been taken this last decade by reconsideration of haplo-identical family donors. Initially associated with very disappointing results, mainly because of unacceptably high incidences of graft rejection and severe graft-versus host disease (GVHD), haplo-identical transplants have benefited from advances in the effective *ex vivo* depletion of T-cells or more recently of unmanipulated *in vivo* regulation of T-cells [4]. The latter, also known as T-replete haplo-identical allo-SCT, has been proven to be feasible and has the advantage to be reproducible by all teams worldwide and at a lower cost than other unrelated transplants.

One of the most recognized approaches to perform modern haplotransplant is that established by the pioneering group of Baltimore using early administration of two days of high-dose post-transplant cyclophosphamide (PTCY) after a fludarabine/low dose CY/low-dose total body irradiation RIC regimen with bone marrow (BM) as stem cell source. With this strategy, graft failure is around 13% and very low incidences of severe acute (5% of grade 3–4) or chronic extensive (5%) GVHD are observed [5, 6]. These results are so encouraging that PTCY has also been used as a sole GVHD prophylaxis after myeloablative allotransplants with matched donors [7–9].

Nevertheless, if the Baltimore regimen is now considered a standard-of-care haplo-regimen, relapse remains a matter of concern, especially for myeloid malignancies. More intensive conditioning regimen or replacement of fludarabine by clofarabine, a second generation purine analog with higher anti-leukemic activity, may be of interest for these patients [10, 11] The use of peripheral blood stem cells (PBSC) as a graft source has also been proven to be as efficient as BM without excessive toxicity, especially no increased incidence of GVHD, in the haplo/PTCY setting [12].

Totally non myeloablative, PTCY inhibits alloreactivity in both the host-versus-graft (rejection) and graft-versus-host directions. PTCY selectively kills proliferating alloreactive T-cells while sparing non-alloreactive T-cells (including regulatory T-cells) responsible for immune reconstitution and resistance to infection [4]. However, the influence of PTCY on both early immune reconstitution and outcome after allo-SCT has been poorly studied so far, [13–15] especially in comparison to the standard use of anti-thymocyte globulin (ATG) as GVHD prophylaxis for matched RIC allo-SCT, especially in Europe [16].

Here we compared the impact on early outcomes and immune recovery of PTCY vs ATG for RIC PBSC allo-SCT.

## RESULTS

### Patients and Samples

Between August 2014 and February 2017, 45 patients were included in the study. There were 30 patients enrolled in the PTCY group, 10 with matched and 20 with haplo-identical donors, and 15 patients in the ATG group, all but one with matched donors. Median age for the whole cohort was 63 years (range: 24–72). There were no differences between both groups in terms of gender, median age, type and status of diseases, previous allograft, type of RIC regimens (clofarabine vs fludarabine-based), median stem cells dose infused or immune status before the graft. Characteristics of RIC regimens are given in Table 1 and of patients in Table 2. Blood samples were collected for each patient before starting the conditioning regimen, then 3 times per week at 2 days intervals from day +0 until day+30, then at days +60 and +90/100. Thus, day +0, +30, +60 and +90/100 were fixed days and a median of 9 samples were collected between day +0 and day +30 for each patient. A total of 583 samples were analyzed: 552 from patients (44 before start of conditioning, 508 between days 0–100), 26 from graft samples and 5 from healthy controls. The number of samples analyzed between days 0–30 (PTCY: 269 samples [median per patient *n* = 9]) vs ATG: 121 samples [median per patient *n* = 8]), at day +30 (PTCY *n* = 30 vs ATG: *n* = 15), day +60 (PTCY *n* = 23 vs ATG *n* = 14) and day +90/100 (PTCY *n* = 21 vs ATG *n* = 15) were similar between both groups.

**Table 1:** Details of reduced-intensity conditioning regimens, donor type and graft-versus-host disease prophylaxis

PTCY group *N* = 30 Matched donors *n* = 10 Haploidentical donors *n* = 20	ATG group *N* = 15 All matched donors, except 1 (9/10)
Fludarabine or clofarabine 30 mg/m^2^/day day-6 to day-2 Cyclophosphamide 14.5 mg/Kg day-6 Low dose total body irradiation 2 grays day-1	Fludarabine or clofarabine 30 mg/m^2^/day day-6 to day-2 Busulfan IV 3.4 mg/kg/day day-3 and day-2
PTCY 50 mg/kg/day day +3 (*n* ***=*** 5) or days +3, +4 (*n* ***=*** 15)	ATG 2.5 mg/kg/day day-1 (*n* ***=*** 5) or days −2 and −1 (*n* ***=*** 10)
GVHD prophylaxis CsA + MMF in all cases Beginning after last dose of PTCY	GVHD prophylaxis CsA (sibling donor) or CsA + MMF (MUD, 9/10) Beginning day-3.
G-CSF systematically administered from day+1	G-CSF not systematically administered

Abbreviations: RIC: reduced-intensity conditioning; GVHD: graft-versus-host disease; PTCY: post-transplant Cyclophosphamide; ATG: anti-thymocyte globulin; CsA: cyclosporine A; MMF: mycophenolate mofetil; G-CSF: granulocyte colony stimulating factor; mud: matched unrelated donor.

**Table 2:** Characteristics of patients, donors, graft and long-term outcomes

	PTCY group *N* = 30	ATG group *N* = 15	*P* value
**Gender : male**	23 (77%)	8 (53%)	NS
**Median age: years (range)**	62 (24–72)	65 (32–72)	NS
**Diseases**			
Myelodysplastic syndrome	4	3	
Acute Myeloid Leukemia	11	7	
Acute Lymphoblastic Leukemia (B/T)	2 (1/1)	2 (2/0)	
Lymphoma (T)	1		
Hodgkin disease	3	2	
Myelofibrosis (primary/secondary)	7 (5/2)		
Chronic Lymphocytic Leukemia	1		
Chronic myeloid leukemia	1	1	
Myeloid/lymphoid	23 (77%)/7 (23%)	10 (67%)/5 (33%)	NS
**Status at transplant**			
CR1/CR2	13/2 (43% CR1)	9/2 (60% CR1)	NS
PR2/PR3/PR4	2/2/1 (23%)	0/3/0 (20%)	
Active	10 (33%)	1 (7%)	NS
**Previous allograft: yes**	6 (20%)	1 (7%)	NS
Reduced Intensity Conditioning (RIC) regimen^*^			
Fludarabine-based+PTCY (1 day/2 days)	15 (5/10)		
Clofarabine-based +PTCY 2 days	15		
Fludarabine-based +ATG 2 days		5	
Clofarabine-based +ATG (1 day/2 days)		10 (5/5)	
Clofarabine-based regimen	15 (50%)	10 (66%)	NS
**Type of donors**			
Sibling (brother/sister)	4 (1/3)	8 (2/6)	
Matched unrelated (male/female)	6 (5/1)	6 (5/1)	
9/10 unrelated mis-matched (male)		1	
Haplo-identical	20		
(brother/sister/son/daughter/nephew/mother/father)	(3/4/7/3/1/1/1)		
Median CD34+ graft cells infused (10^*^6/Kg)	8 (3.9–22)	6.59 (4.57–10.02)	NS
Median CD3+ graft cells infused (10^*^7/Kg)	24 (11–41)	23 (8.6–37)	NS
**Immune status before graft: /mm3 (25%/75% percentile)**			
Median B cells	24 (2–76)	11 (0.16–36)	NS
Median CD4^+^ T cells	411 (190–638)	267 (71–398)	NS
Median CD8^+^ T cells	251 (92–401)	347 (129–509)	NS
Median α/β T cells	603 (381–1001)	606 (275–941)	NS
Median γ/δ T cells	27 (8.3–53)	18 (5.2–42)	NS
Median iNKT cells	49 (8.5–64)	22 (8.1–67)	NS
Median NK cells	67 (34–120)	50 (24–179)	NS
Median Tregs	13 (7.5–34)	8.2 (3.3–16)	NS
Median Monocytes	734 (299–1028)	659 (255–994)	NS
**Long term outcomes**			
Disease-Free Survival at one year (%)	80 ± 7	60 ± 13	NS
Disease-Free Survival at two years (%)	63 ± 10	60 ± 13	NS
Overall Survival at one year (%)	90 ± 6	73 ± 11	NS
Overall Survival at two years (%)	79 ± 9	73 ± 11	NS
Relapse incidence (%)	17%	33%	NS
Median time of relapse: months (range)	5 (4–11)	6 (3, 5–10)	NS

Abbreviations: PTCY: post-transplant cyclophosphamide; ATG: anti-thymocyte globuline; CR: complete remission; PR: partial remission. NS: not significant. ^*^For details regarding RIC regimens, see Table 1.

### Comparison of early outcomes

Only engrafted patients were considered in this study. The median time of neutrophils recovery was similar between both groups (PTCY: 18 days [range: 10–30] vs ATG 17 days [range: 13–25]), while platelets recovery was significantly delayed in the PTCY group (median 31 days [range: 9–104] vs ATG 12 days [range: 10–39], *p* = 0.002). Median whole blood donor chimerism was similar at day +30 (PTCY: 100% [range: 78–100] vs ATG 98% [range: 58.3–100], day +60 (100% [range: 68–100] vs ATG 99% [range: 44–100] and day +90/100 (100% [range: 19–100] vs ATG 100% [range: 62–100]) between the two groups. Conversely, CD3+ donor chimerism was significantly higher in the PTCY group at day +60 (100% [range: 73–100] vs 87% [range: 0–100], *p* = 0.0008) and day +90/100 (100% [range: 79–100] vs ATG 92% [range: 0–100], *p* = 0.0008).

Between days 0–90/100, the rate of HHV-6 reactivations was significantly increased in the PTCY group (53% vs 7%, *p* **=** 0.0017) while EBV reactivations were significantly associated with the ATG group (73% vs 20%, *p* = 0.0003). HHV-6 reactivations were significantly higher (*p* = 0.008) for patients receiving PTCY and having haploidentical donors (*n* = 14/20, 70%) vs patients receiving PTCY but having matched donors (*n* = 2/10, 20%). Moreover, pre-emptive treatment by rituximab for EBV reactivation was performed in 5 patients in the ATG group vs 0 in the PTCY group. Similar rates of CMV (all treated) (PTCY 27% vs ATG 40%, *p* = NS) and BKv (PTCY: 15% vs ATG: 20%) reactivations were noted between both groups. Only one adenovirus reactivation was detected, in the PTCY group. Interestingly, patients receiving only 1 day of PTCY did not develop any viral infection, except one case documented with moderate EBV reactivation, with no need of therapy. Incidences of acute grade 2–4 and grade 3–4 GVHD, were similar between both groups (PTCY: 47% and 10% vs ATG: 47% and 20%). None of the patients had relapsed or died before day 100. At last follow-up (May 2017), 5 (17%) patients had relapsed in the PTCY group (2 with matched donors and 3 with haplo-identical donors) vs 5 (33%) in the ATG group. Also, 7 (23%) and 5 (33%) have died in the former and the latter groups, respectively. All patients who died in the PTCY group have been transplanted with a haplo-identical donor except one, the causes of death being grade 4 gut acute GVHD in 2, dermatomyositis in 1, rhinovirus infection in 1 and relapse in 3. Causes of death in the ATG group were acute distress respiratory syndrome in 2, cardiac insufficiency in 1, sepsis in 1 and relapse in 1. Long-term outcomes are given in Table 2.

### Comparison of immune recovery between both groups (PTCY vs ATG)

**Figure 1:**
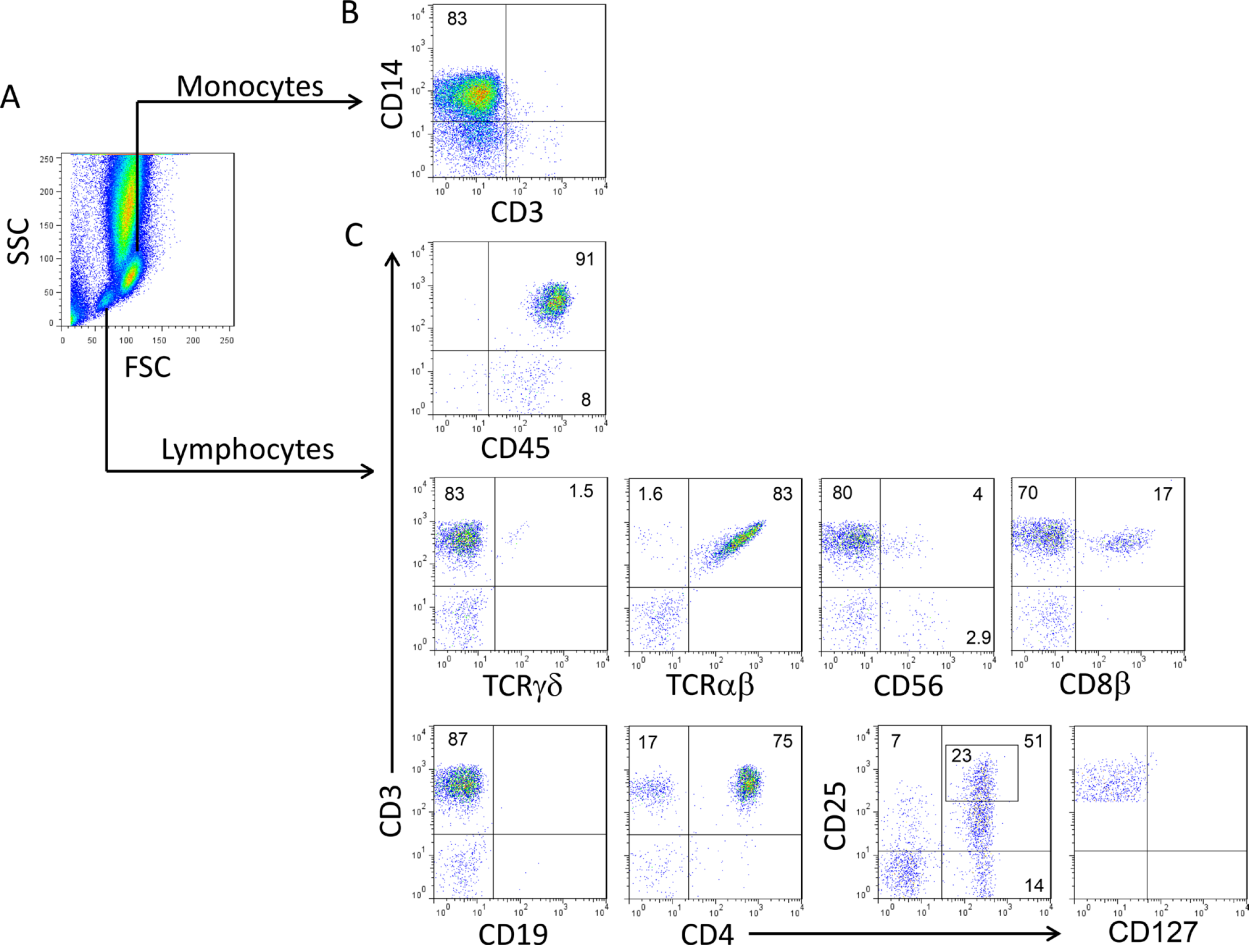
Peripheral blood cell populations evaluated by flow cytometry between days 0 and +90/100 after allotransplant Representative density plots illustrating the gating strategy to investigate the major cell populations in peripheral blood by flow cytometry. (**A**) Monocytes and lymphocytes were identified using a SCC/FSC cell gating strategy, (**B**) then monocytes (CD3^−^ CD14^+^) were considered in the monocyte gate (**C**) and finally the following cell populations were considered among lymphocytes: gamma/delta (γ/δ)T-cells (CD3^+^/γ/δ^+^), alpha/beta (α/β)T-cells (CD3^+^/α/β^+^), NK T-cells (NKT as CD3^+^/CD56^+^); NK cells (CD3^−^/CD56^+^), CD8^+^ T-cells (CD3^+^/CD8β^+^), B-cells (CD3^−^/CD19^+^), CD4^+^ T-cells (CD3^+^/CD4^+^), regulatory (Tregs) T-cells (CD3^+^/CD4^+^/CD25^high^/CD127^low/−^). The lymphocyte population was validated by CD45 expression.

**Figure 2:**
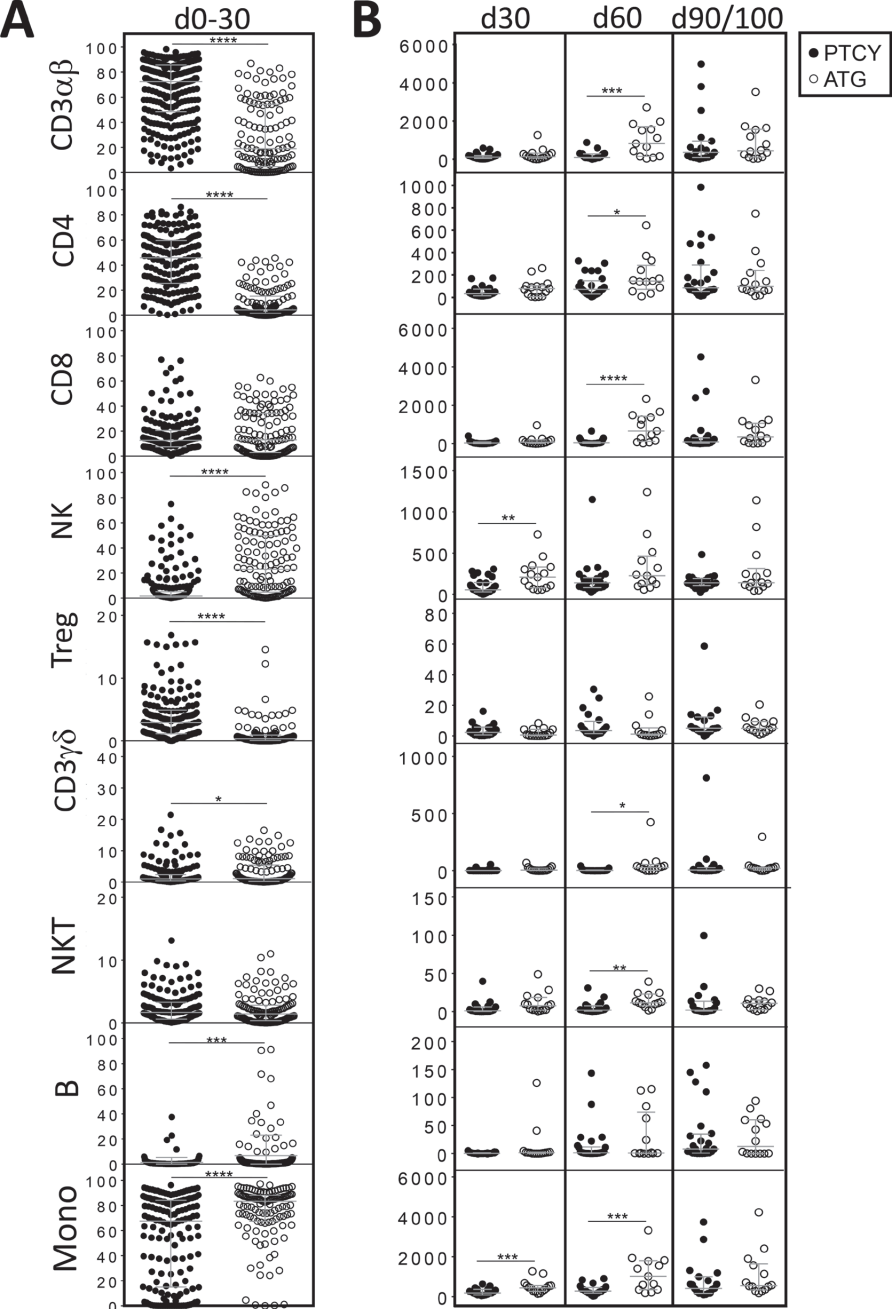
Comparison of numerical immune reconstitution between PTCY and ATG groups considering the whole cohort (**A**) Scatter plots representing the studied cell frequencies (CD3αβ, CD4, CD8β, NK, Treg, CD3γδ, NKT, B and monocytes) observed in peripheral blood of patients between day 0 and day 30. Results are represented as medians with interquartile ranges. (**B**) Scatter plots representing the absolute counts of studied cell populations in peripheral blood of patients at day 30, 60 and 90/100 since HSCT. Results are represented as medians with interquartile ranges. Statistical significance (^*^p < 0.05, ^**^p < 0.01, ^***^p < 0.001, ^****^p < 0.0001) was determined between two groups using the unpaired *t*-test and was determined between more than two groups using the one-way ANOVA test.

For all studied samples, we identified α/β T-cells, CD8^+^ T-cells, CD4^+^ T-cells, NK cells, NK T-cells, γ/δ T-cells, Tregs, B-cells and monocytes by flow cytometry (Figure 1). Between days 0–30, the percentages of α/β T-cells, Tregs and CD4^+^ T-cells were significantly higher in the PTCY group while it was the case for B and NK cells, γ/δ T-cells and monocytes in the ATG group (Figure 2A). Considering absolute numbers, ATG was associated with higher median counts of NK and monocytes at day +30 and higher median counts of α/β and γ/δ T-cells, CD4+ and CD8+ T-cells, NK T-cells and monocytes at day+60 (Figure 2B). CD4^+^ T-cells increased progressively after transplant but did not recover to the clinical laboratory–defined normal range (500–1200 cells/μl) at day+90/100 in both groups (Figure 2B). Profound B-cells (normal range: 100–400 cells/μl) lymphopenia was observed in both groups (Figure 2B). NK cells (normal range: 100–400 cells/μl) and monocytes (normal range: 150–900 cells/μl) recoveries were rapid and similar for both groups reaching normal counts (100–400 cells/μl) as soon as day+30 (Figure 2B). CD8^+^ T-cells counts remained low in the PTCY group while it was not the case for the ATG group where CD8 reached normal range (300–700 cells/μl) at day+60 (Figure 2B).

### Influence of the type of donor

**Figure 3:**
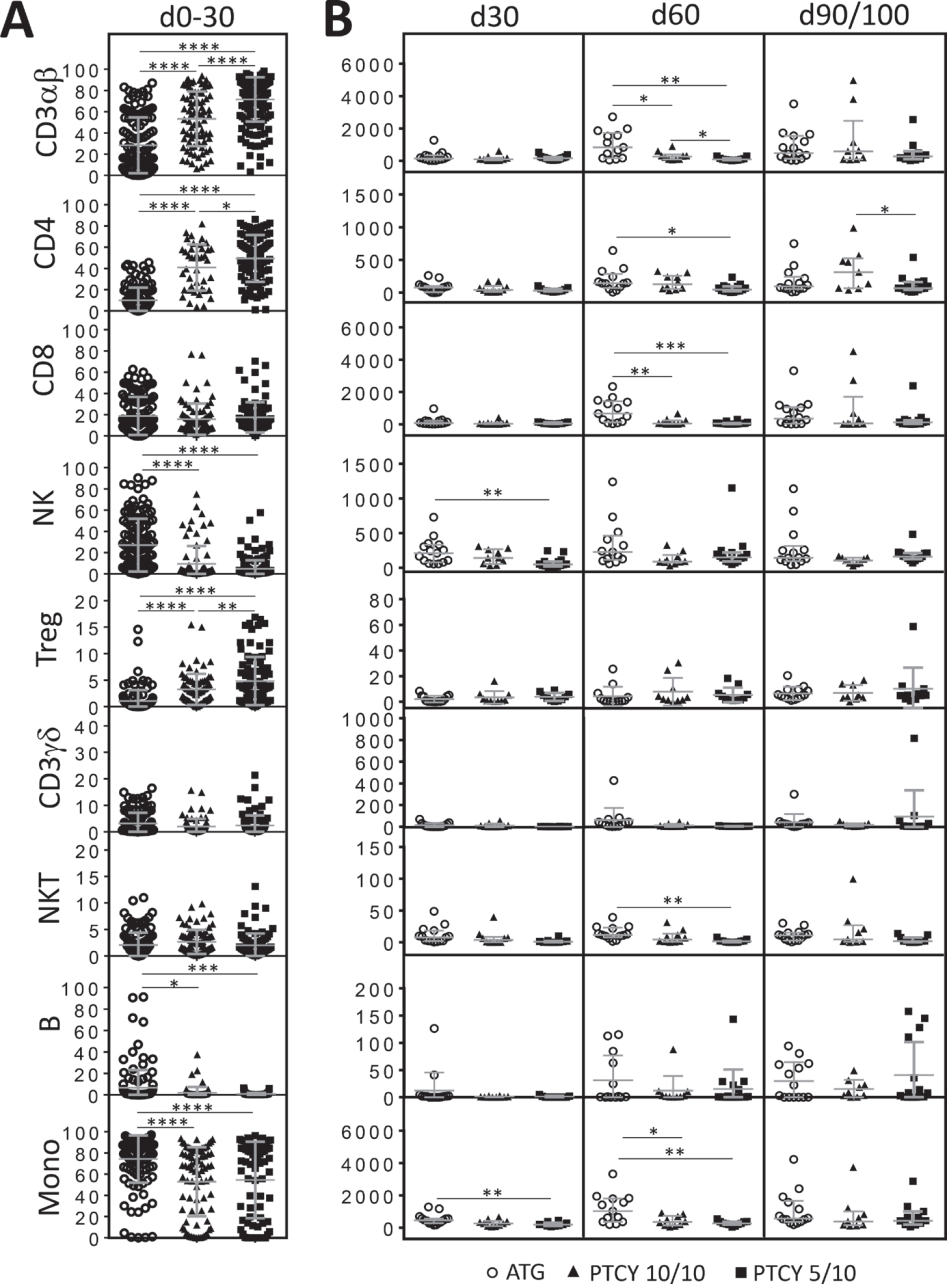
Comparison of numerical immune reconstitution between haplo donors/PTCY, matched donors/PTCY and ATG groups (**A**) Scatter plots representing the studied cell frequencies (CD3αβ, CD4, CD8β, NK, Treg, CD3γδ, NKT, B and monocytes) observed in peripheral blood of patients between day 0 and day 30. Results are represented as median with interquartile range. (**B**) Scatter plots representing the absolute counts of studied cell populations in peripheral blood of patients at day 30, 60 and 90/100 since HSCT. Results are represented as medians with interquartile ranges. Statistical significance (^*^p < 0.05, ^**^p < 0.01, ^***^p < 0.001, ^****^p < 0.0001) was determined between the two groups using the unpaired *t*-test and was determined between more than two groups using the one-way ANOVA test.

When considering only matched donors (PTCY *n* = 10, ATG *n* = 15), between days 0–30, percentages of α/β T-cells, Tregs and CD4^+^ T-cells were significantly higher in the PTCY group while it was the case for NK cells, B-cells and monocytes in the ATG group (Figure 3A). At day +60, higher median absolute numbers of α/β T-cells, CD8+ T-cells and monocytes were documented in the ATG group. When considering only PTCY patients, between days 0–30, significantly higher percentages of α/β T-cells, Tregs and CD4^+^ T-cells were documented for patients with haplo-identical donors (Figure 3A). Conversely, patients with matched donors had significantly higher median absolute numbers of α/β T-cells at day +60 and of CD4+ T-cells at day+90/100, (Figure 3B). When considering only PTCY patients with haplo-identical donors, comparison of both groups (ATG vs PTCY) showed, between days 0–30, significantly higher percentages of α/β T-cells, Tregs and CD4^+^ T-cells for PTCY patients and higher percentages of NK cells, B-cells and monocytes for ATG patients (Figure 3A). ATG patients also had, significantly higher median absolute numbers of NK cells and monocytes at day +30, and of α/β T-cells, CD4+ and CD8+ T-cells, NK T-cells and monocytes at day +60 (Figure 3B).

### Influence of clofarabine

**Figure 4:**
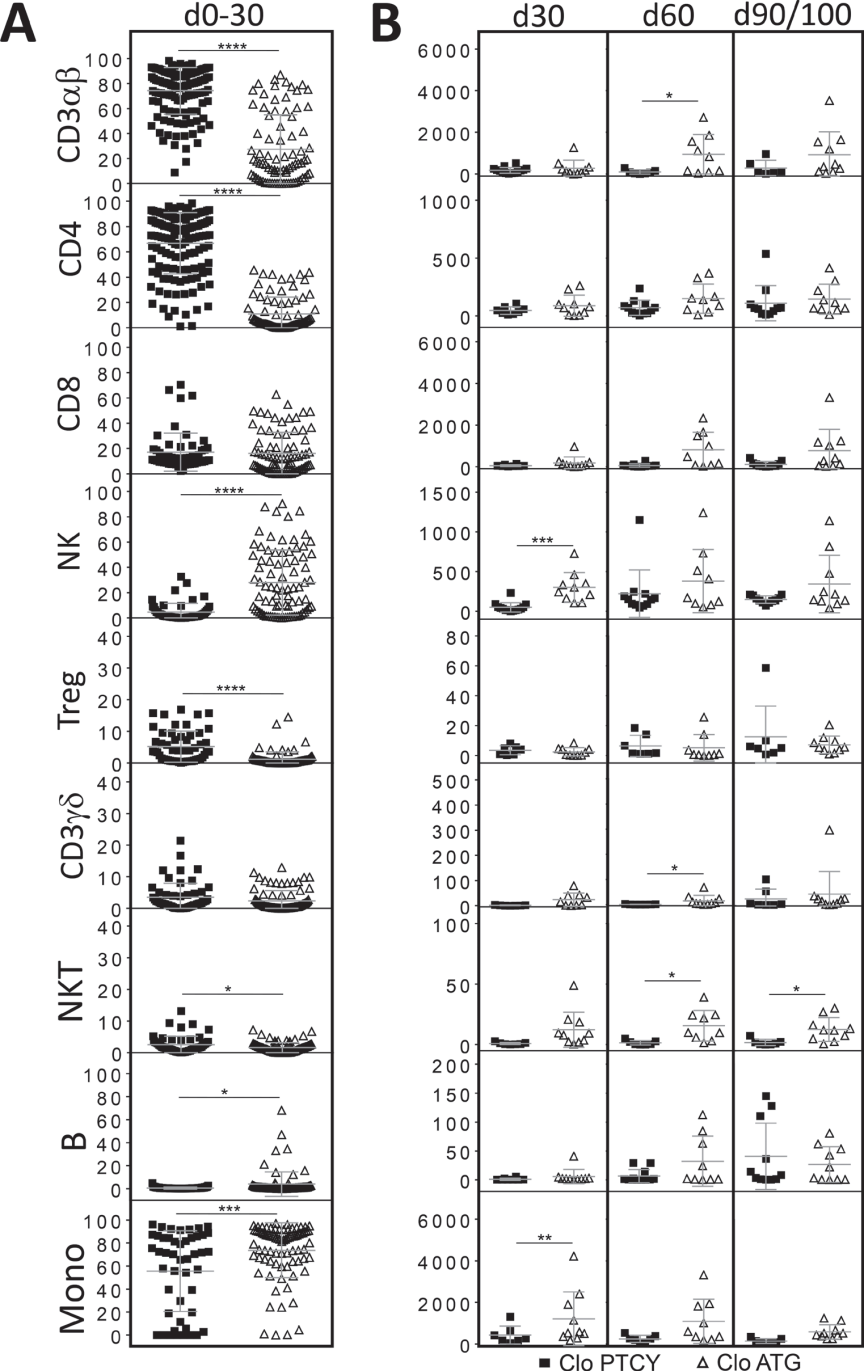
Comparison of numerical immune reconstitution between PTCY and ATG subgroups considering only patients receiving clofarabine-based regimens (**A**) Scatter plots representing the studied cell frequencies (CD3αβ, CD4, CD8β, NK, Treg, CD3γδ, NKT, B and monocytes) observed in peripheral blood of patients between day 0 and day 30. Results are represented as medians with interquartile ranges. (**B**) Scatter plots representing the absolute counts of studied cell populations in peripheral blood of patients at day 30, 60 and 90/100 since HSCT. Results are represented as medians with interquartile ranges. Statistical significance (^*^p < 0.05, ^**^p < 0.01, ^***^p < 0.001, ^****^p < 0.0001) was determined between two groups using the unpaired *t*-test and was determined between more than two groups using the one-way ANOVA test.

When considering only patients receiving clofarabine-based regimens (PTCY *n* = 15, ATG *n* = 10), between days 0–30, the percentages of α/β T-cells, Tregs, CD4^+^ T-cells and NK T-cells were significantly higher in the PTCY group while it was the case for B and NK cells, and monocytes in the ATG group (Figure 4A). Considering absolute numbers, ATG patients had higher median counts of NK and monocytes at day +30, of α/β and γ/δ T-cells and NK T-cells at day+60 and of NK T-cells at day+90/100 (Figure 4B).

## DISCUSSION

This prospective study aimed to clarify the influence of PTCY on early outcome and immune reconstitution after PBSC RIC allo-SCT, either using matched or haploidentical donors. For this purpose, a cohort of patients transplanted with matched donors and receiving ATG as GVHD prophylaxis were used for comparison. Although the number of patients is small, we could observe some strong differences in terms of hematopoietic recovery, chimerism, viral infections and immune reconstitution following these two types of transplant.

So far, retrospective studies have shown that the newly developed haplo-transplantation procedures can provide similar or sometimes better survivals when compared to transplants using sibling, matched or one antigen mismatched (9/10) unrelated donors or cord blood (CB) as stem cell source [20–27]. This seems to be also the case when considering the use of PTCY for matched vs haplo-identical donors, as shown recently [25]. Although our study was not designed to compare long-term outcomes, relapse and death rates were similar between both groups.

Our data were focalized on early outcomes after ATG vs PTCY transplant. We showed that higher CD3^+^ donor chimerism, higher HHV-6 and lower EBV reactivations were observed with PTCY. This could be related to the particular immune reconstitution seen after PTCY administration. From our study, we were able also to identify some clinical advantages and drawbacks of PTCY. If delayed platelets recovery is a hallmark of PTCY, [25, 28] higher donor CD3 chimerism was observed at days +60 and +90/100, a result which may have some importance in terms of preventing relapse [29]. This pleads for the use of PTCY not only in the matched donor setting but also in a CB context where it is known that the use of ATG is deleterious [30]. As reported before, [31] higher HHV-6 reactivation was observed in the PTCY group together with less EBV reactivations and similar CMV reactivations, a profile which is characteristic of ATG-free CB transplant [18] High donor/recipient HLA mismatches (as for CB transplant) may explain these results since HHV-6 reactivation was significantly higher for PTCY patients with haplo-identical vs matched donors. This suggests that PTCY itself may not be associated with higher viral infections after transplant, [15] as demonstrated especially when it is administered only one day in patients with a matched donor. Indeed, no viral infection but one (with no need of pre-emptive therapy) was documented in this subgroup and the use of lower PTCY doses has been already reported, associated to less BKv hemorrhagic cystitis [32]. Also HHV-6 reactivations were not predicting of CMV reactivations, as described recently [33].

As already mentioned, immune reconstitution has been poorly studied when considering allotransplant using PTCY, [13–15] and no comparison with matched/ATG transplants has been reported so far. Indeed, most data result from haploidentical transplants using ATG as GVHD prophylaxis, showing some favorable impact on outcomes for early lymphocytes and NK cells recoveries [34–41]. No such data are yet available in the PTCY setting.

Here, we were able to document during the first 30 days, a period of profound lymphopenia, the percentages of various cell subsets, showing some early survival advantage for CD4^+^ T-cells, Tregs and α/β T-cells in the PTCY group while it was the case for γ/δ T-cells, NK cells and monocytes in the ATG group. This was not influenced by the use of mismatched donors as similar results were obtained when considering only matched or haplo-identical donors of the PTCY group by comparison to the ATG group, suggesting a direct effect of the particular GVHD prophylaxis used in each cohort. Persistence of activated Tregs that survive PTCY in the first 1–2 months is the hallmark of haplo-identical transplant using bone marrow as stem cell source [15]. The fact that it was also the case here, using PBSC as graft source, is not surprising as Tregs, the cells responsible for tolerance after transplant, are known after allogeneic stimulation to increase their expression of aldehyde dehydrogenase, the major *in vivo* mechanism of cyclophosphamide detoxification, thereby becoming cyclophosphamide resistant [15].

In PTCY conditioning regimen, cyclophosphamide targets proliferating cells as NK and CD8^+^ T cells but maintains CD4^+^ T cells and Treg in significant numbers. In ATG conditioning regimen, CD3^+^ T are mainly targeted in contrast to NK cells which are maintained in frequency and high number during the first month. From day +60, the effect of cyclophosphamide on the overall T cell compartment is observed and comparable immune T cell reconstitution is achieved for both groups. This observation raises the question of the beneficial and adverse impacts of CD4^+^ T cells on clinical outcome during the first month with the PTCY conditioning regimen.

When considering reconstitution in terms of absolute numbers, if NK and monocytes recoveries reached normal counts as soon as day+30 in both groups, higher counts for these two populations were observed at days +30 and /or day+60 in the ATG group. This was not influenced by the use of mismatched donors as similar results were obtained when considering only matched or haplo-identical donors for the PTCY group in comparison to ATG, suggesting once again a direct effect of the particular GVHD prophylaxis used in each cohort. This may be of importance as higher early monocytes counts have been shown to significantly influence favorably outcomes [42, 43]. However, these differences were not observed in the literature when comparing matched transplants vs haploidentical transplants using ATG as GVHD prophylaxis, [36] suggesting an important role for the latter regarding immune reconstitution.

Higher counts of CD4^+^ and CD8^+^ T-cells, α/β and γ/δ T-cells, NKT and monocytes were also observed in the ATG group at day+60. When considering only matched donors, only higher counts of α/β, CD8^+^ T-cells and monocytes were found in the ATG group vs the PTCY group. Thus, haploidentical donors may also influence immune reconstitution, besides the GVHD prophylaxis used. Indeed, when considering only PTCY patients, between days 0–30, percentages of CD4^+^ T-cells, α/β T-cells and Tregs were significantly higher for patients with haplo-identical donors but at day+60 and +90/100, lower counts of α/β T calls and CD4+ T-cells were noted in the same cohort.

Another originality of our study was that clofarabine was tested both in the PTCY and the ATG groups as part of the conditioning regimen for myeloid malignancies. Clofarabine was responsible for an early survival advantage of iNK T-cells in the PTCY group while it abrogated the early survival advantage of γ/δ T-cells in the ATG group. Higher early post-transplant iNK T-cells recovery has been associated with lower acute GVHD and non-relapse mortality incidences as well as better overall survival [44], Also, it is now well established that γ/δ T-cells exhibit cytotoxicity toward a large range of tumors *in vitro* or *in vivo* and that tumor recognition by these cells requires neither antigen uptake, nor MHC class I or II expression, allowing for a very rapid response to the immune challenge [45]. Thus, use of clofarabine may have some importance in terms of relapse prevention in the haplo/PTCY setting and may be part of the explanation for a higher antileukemic activity compared to fludarabine as part of the conditioning regimen [46].

Finally, if PTCY seems to be associated with a better survival than ATG as GVHD prophylaxis when considering haplo-identical allografts for acute myeloid leukemia patients, [47] it remains undetermined if it will be better to consider PTCY or ATG as GVHD prophylaxis for patients with matched donors. The question remains open, as well as that of using both ATG + PTCY as GVHD prophylaxis. Dose or drug modifications within the RIC regimen in both groups may be envisioned to favor some cell population recoveries after allo-SCT which may be of interest in terms of better relapse prevention or tolerance.

In conclusion, strong differences exist in term of outcome and early immune recovery when using PTCY or ATG as part of the GHVD prophylaxis for RIC PBSC allo-SCT. Larger studies are needed to confirm these preliminary but first reported results in the PTCY setting.

## MATERIALS AND METHODS

### Study design and data collection

This was a prospective study conducted at Nantes University Hospital with the aim to compare early outcomes and immune recovery until day+90/100 post-transplant between patients receiving PTCY or ATG as part of the GVHD prophylaxis after a RIC PBSC allo-SCT. Secondary objectives were to study the impact on immune reconstitution of haplo-identical vs matched donors and of clofarabine as part of the RIC regimen. All RIC regimens and donors were permitted for a total of 30 engrafted patients in the PTCY group and 15 in the ATG group. Fludarabine-based RIC regimens were mainly used for lymphoid malignancies and clofarabine-based RIC regimens for myeloid malignancies [11]. The source of graft was PBSC for all cases. Details of each regimen, GVHD prophylaxis and type of donor are given in Table 1. A declaration of preparation and conservation of this biocollection (DC-2014-2340) has been forwarded to French Research’s Minister and has received agreement from the IRB (2015- DC-1). The study was approved by the Ethic Review Board of of Nantes University Hospital and all patients provided informed consent.

### Outcomes to be studied

Neutrophil recovery was defined as the first of 3 consecutive days with a measured absolute neutrophil count of > 0.5 × 10^9^/L. Platelet recovery was defined as the first of 3 consecutive days with a platelet count of > 50 ×10^9^ /L without transfusion support. Whole blood and CD3 T-cells donor chimerisms were evaluated at days +30, +60 and +90/100, according to standard methods [17]. Also, viral reactivations (Cytomegalovirus, CMV, Epstein-Barr virus, EBV, and Human herpesvirus 6 [HHV-6], adenovirus and BKv virus) were studied twice a week until days 0–90/100 according to standard methods [18]. Rates of relapse, overall and disease free survivals at day +90/100 were calculated. Acute GVHD was graded according to standard criteria [19].

### Immunophenotypic analysis by flow cytometry

Blood samples were collected for each patient before initiation of the conditioning regimen then 3 times per week at 2 days intervals from day +0 until day +30, then at days +60 and +90/100. Briefly, samples were used directly after red blood cells lysis following 10 minutes incubation with the Erythrocyte-Lysing Reagent without Fixative, EasyLyse™ (Dako France, Les Ulis, France). Immunophenotype were assessed in four-color flow cytometry using the following mouse anti-human monoclonal antibodies: CD4-PE (13B8.2), pan α/β-PE (IP26A), pan γ/δ-FITC (IMMU510) (Beckman Coulter, Miami, FL), CD19-FITC (SJ25C1), CD3-PerCp (SK7), CD4-FITC (RPA-T4), CD8β-APC (25T8-5H7), CD14-APC (MSE2), CD25-FITC (2A3), , CD45-APC (HI30), CD56-APC (B159 (BD Biosciences), and CD127-APC (40131, R&D Systems Europe, Lille, France). All flow cytometry data were collected using a FACSCalibur (BD Biosciences, San Jose, CA), and analyzed using Flowjo 7.6.1 software (TreeStar, Ashland, OR). For all samples, monocytes and lymphocytes were identified using a SCC/FSC cell gating strategy, then the following cell populations were considered: gamma/delta (γ/δ)T-cells (CD3^+^/γ/δ^+^), alpha/beta (α/β)T-cells (CD3^+^/α/β^+^), NK T-cells (NKT as CD3^+^/CD56^+^); NK cells (CD3^−^/CD56^+^), CD8^+^ T-cells (CD3^+^/CD8β^+^), B-cells (CD3^−^/CD19^+^), CD4^+^ T-cells (CD3^+^/CD4^+^), regulatory (Tregs) T-cells (CD3^+^/CD4^+^/CD25^high^/CD127^low/−^), and finally monocytes (CD3^−^ CD14^+^) in a monocyte gate. The lymphocyte population was validated by CD45 expression (Figure 1). Only the median percentage (%) compared to total lymphocytes was considered for all lymphocytes subsets between days 0–30 because of profound lymphopenia in patients. Thereafter, both % and median absolute numbers were considered for samples collected at days +30, +60 and +90. Absolute numbers of cell subsets were calculated by multiplying the total nucleated cells count by the total percentage of each considered cell population as determined by flow cytometry from the respective whole sample. Absolute numbers were expressed as cells/μl. Analyses of the cell populations of the graft samples were not performed here. Five healthy donors were used after they signed informed consent as controls.

### Statistical analyses

Results were given as medians with (25%/75%) percentiles. Univariate comparisons were performed by the Student’s *t*-test and comparisons of multiple groups by the one-way ANOVA test, both using the GraphPad Prism v6.0 software (San Diego, CA). *P* values < 0.05 were considered statistically significant.

## Abbreviations

PTCY: post-transplant cyclophosphamide
allo-SCT: allogeneic stem cell transplantation
ATG: anti-thymocyte globulin
GVHD: graft-versus-host disease
RIC: reduced-intensity conditioning
PBSC: peripheral blood stem cell
BM: bone marrow
